# Research Progress on Asphalt–Aggregate Adhesion Suffered from a Salt-Enriched Environment

**DOI:** 10.3390/ma19010192

**Published:** 2026-01-05

**Authors:** Yue Liu, Wei Deng, Linwei Peng, Hao Lai, Youjie Zong, Mingfeng Chang, Rui Xiong

**Affiliations:** 1 School of Materials Science and Engineering, Chang’an University, Xi’an 710061, Chinadengwei@chd.edu.cn (W.D.); 2017903772@chd.edu.cn (H.L.); mfchang99@126.com (M.C.); 2 College of Materials Science and Engineering, Xi’an University of Architecture and Technology, Xi’an 710055, China

**Keywords:** salt-rich environment, asphalt pavement, asphalt–aggregate adhesion, evaluation method, improvement measure

## Abstract

Salt permeation erosion is a key factor leading to the deterioration of service performance and shortening the lifespan of asphalt pavement in salt-rich areas. In this environment, the combined action of water and salt accelerates the decline in the asphalt–aggregate interface, leading to distress, such as raveling and loosening, which severely limit pavement durability. The authors systematically reviewed the research progress on asphalt–aggregate adhesion in a saline corrosion environment and discussed the complex mechanisms of adhesion degradation driven by intrinsic factors, including aggregate chemical properties, surface morphology, asphalt components, and polarity, as well as environmental factors, such as moisture, salt, and temperature. We also summarized multi-scale evaluation methods, including conventional macroscopic tests and molecular dynamics simulations, and revealed the damage evolution patterns caused by the coupled effects of water, salt, heat, and mechanical forces. Based on this, the effectiveness of technical approaches, such as asphalt modification and aggregate modification, is explored. Addressing the current insufficiency in research on asphalt adhesion under complex conditions in salt-rich areas, this study highlights the necessity for further research on mechanisms of multi-environment interactions, composite salt erosion simulation, development of novel anti-salt erosion materials, and intelligent monitoring and early warning, aiming to provide a theoretical basis and technical support for the weather-resistant design and long-term service of asphalt pavement in salt-rich regions.

## 1. Introduction

**Figure 1:**
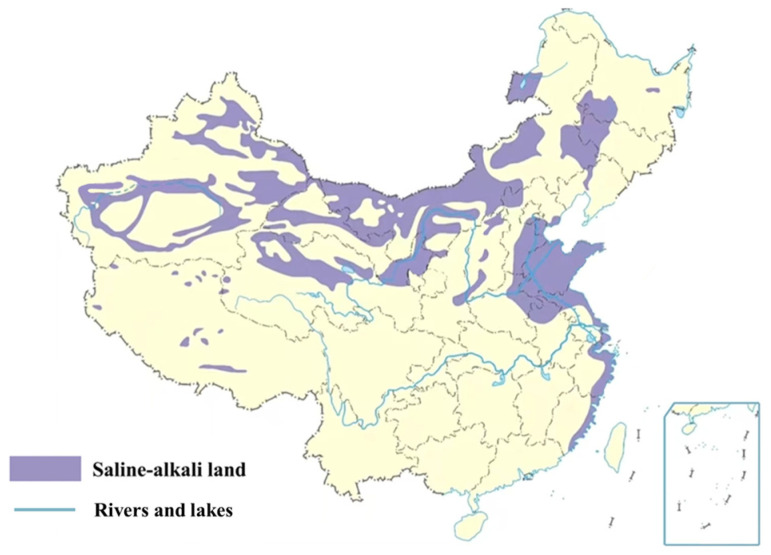
Schematic map of high-salinity areas in China.

Asphalt pavement is widely used due to its good driving comfort and economic benefits. However, during the service life of asphalt pavement, it is subjected to long-term driving loads and direct effects from complex environments, resulting in a continuous decline in its quality of use. As the primary construction material for asphalt pavements, the durability of asphalt mixtures remains a significant scientific concern within the global road engineering community [1,2,3]. Salt-rich regions are widely distributed across China (as illustrated in Figure 1), covering approximately 10% of the nation’s total land area. According to the Third National Land Survey, saline–alkali soils, which are dominated by sulphates and chlorides, cover 96.1% of arid and semi-arid regions in central and western China and are extensively distributed across Xinjiang, Qinghai, Inner Mongolia, and Ningxia. The remaining areas comprise carbonate-dominated soda saline–alkali soils in the northeast and chloride-dominated coastal saline–alkali soils along the eastern coastline [4,5].

**Figure 2:**
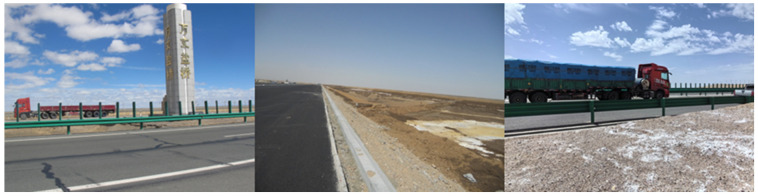
Salt erosion damage conditions of asphalt pavement materials under multi-factor coupling effects (photo taken by the author R.X.).

Whether in coastal regions plagued by frequent sea fog and high salt-laden humidity or in inland salt lakes and saline-affected soils, naturally concentrated saline media infiltrate asphalt pavement surfaces through various pathways, gradually penetrating the surface structure and materials. This occurs via the following: ① salt migration from subgrade material eroding surface layers due to seepage, temperature gradients, and capillary rise; ② wind-blown salt particles and surface salt deposits that form solutions during rainfall and enter the pavement through cracks or voids; ③ in Salt Lake Road areas, salt particles carried by vehicle tires accumulate on the road surface; and ④ salt gradually permeates into the asphalt mixture as snow melts. Coupled with water temperature fluctuations and load effects, these issues frequently cause asphalt pavement deterioration and shorten its service life [6,7]. Moreover, during winter, many roads employ the method of spreading de-icing salts to rapidly eliminate traffic disruptions caused by snow and ice accumulation on asphalt surfaces, thereby ensuring driving safety. Alternatively, innovative approaches, such as incorporating salt-containing de-icing and anti-icing materials into the mix or using salt-retaining asphalt mixtures, are adopted to address this issue [8,9,10,11,12,13]. Engineering environments, such as saline soils and winter de-icing salt applications (as illustrated in Figure 2), exert persistent detrimental effects on asphalt pavement material properties through salt contamination [14]. Concurrently, in salt-rich regions, asphalt pavements frequently endure repeated wet–dry cycles, freeze–thaw cycles, or salt spray exposure, which undoubtedly further degrade pavement durability and service quality, causing traffic accidents and resulting in economic losses [4,15].

In a salt-enriched environment, the synergistic effect of moisture and salt accelerates the destruction of the asphalt–aggregate interface, hastens the aging of the asphalt, weakens the adhesion between the asphalt and the aggregate, and ultimately causes diseases, such as spalling and loosening. This not only affects driving comfort and safety but also significantly impacts the service life of the road surface, resulting in unnecessary economic losses [16,17]. This paper summarizes the influencing factors of asphalt–aggregate adhesion under salt-rich environments, sorting out the research methods and new equipment for asphalt–aggregate adhesion. Measures for improving asphalt–aggregate adhesion are summarized, and prospects are proposed to provide a theoretical basis and technical support for the weathering design and long service life of asphalt pavements in salt-rich areas.

## 2. The Effect of Salt Enrichment on the Adhesion of Asphalt to Aggregate

### 2.1. Aggregate Properties

The influence of aggregates on asphalt–aggregate adhesion can be categorized into two main aspects: physicochemical properties and surface morphology. The chemical properties of aggregates are primarily determined by their chemical composition, which comprises SiO_2_, CaO, CaCO_3_, and minor amounts of Al_2_O_3_. The chemical compositions of various aggregates differ, with the mineral composition and chemical constituents of common road aggregates shown in Table 1. In the 1920s, some scholars began investigating the relationship between the adhesion between asphalt and aggregate and the surface characteristics of the aggregate. It was generally believed that an aggregate with a porous and slightly rough surface promotes adhesion through mechanical interlocking effects [18]. Surface morphology characteristics also vary, and different morphological parameters exert significant differences on their adhesion to asphalt. In the early 21st century, the Aggregate Imaging Measurement System (AIMS) emerged [19]. Subsequently, numerous studies employed this method to establish the relationship between aggregate morphology and mixture properties, optimizing it to more accurately capture aggregate surface texture information while enhancing testing throughput and repeatability.

The adhesion between asphalt and aggregate is significantly influenced by the mineral composition and chemical constituents of the aggregate, with different mineral compositions and chemical compositions exhibiting varying degrees of adhesion with asphalt. Li et al. [20] employed multiple regression analysis to investigate the influence of aggregate mineral properties and moisture conditions on the adhesion of asphalt to the aggregate interface. They concluded that, disregarding moisture conditions, aggregate mineral characteristics constitute the most significant factor affecting interfacial adhesion between asphalt and aggregates. To evaluate the impact of different aggregate mineral compositions on adhesion, Li [21] observed the interface damage after drawing with an electron microscope and found that some asphalt remained in carbonate minerals (calcite, dolomite) at the interface damage, while no asphalt remained in silicate minerals such as quartz. Therefore, it is considered that the adhesion effect between carbonate minerals and asphalt is better than that of silicate minerals. Furthermore, by introducing a new parameter—the interaction parameter B—constructed based on the phase angle (an important parameter reflecting the viscoelastic behavior of asphalt is the phase difference between deformation and stress of asphalt under stress), the study found that dolomite and calcite exhibited superior interaction capabilities with asphalt, whereas sodium feldspar, potassium feldspar, and quartz demonstrated poor interaction capabilities with asphalt. This provides corroboration from another perspective for the conclusions drawn from observing the post-pull-out interfacial failure sites. Zhang et al. [22] investigated the influence of asphalt and aggregate mineral composition on the water sensitivity of asphalt–aggregate bonding. The aggregate composition exerted a significantly greater impact on water sensitivity than the asphalt composition. Clay minerals and anorthite exhibited a pronounced negative effect on water sensitivity, whereas calcite demonstrated a positive influence.

To investigate the adhesion effects between different chemical compositions and asphalt, Andres et al. [23] employed an improved pull-off test to evaluate the adhesion between various oxides and asphalt. Aluminum oxide demonstrated favorable adhesion with asphalt, whereas silicon oxide proved detrimental to interfacial bonding. Du et al. [24] investigated the adhesion and diffusion properties of asphalt on various oxide surfaces, determining that the adhesion energies between asphalt and five oxides followed the order: MgO > CaO > Al_2_O_3_ > Fe_2_O_3_ > SiO_2_. Yin et al. [25] examined the adhesion properties between asphalt and limestone versus granite, concluding that CaCO_3_ in limestone undergoes chemical reactions with certain asphalt components, resulting in stronger adhesion strength than that observed with granite. Cao [26] employed contact angle experiments and surface energy calculations to demonstrate that aggregate properties significantly influence asphalt–aggregate adhesion. Acidic aggregates like granite (predominantly SiO_2_) exhibited poor adhesion to an asphalt binder, whereas alkaline aggregates, such as limestone (predominantly CaCO_3_), demonstrated superior adhesion. Duan et al. [27] selected six commonly used aggregates of differing lithologies. Employing water boiling tests, XRD diffraction analysis, and molecular dynamics simulations, they evaluated the interfacial adhesion between each aggregate and petroleum asphalt. They concluded that the CaO content favors asphalt–aggregate adhesion, whereas the SiO_2_ content exerts a detrimental effect on adhesion properties.

Zeta potential (ζ potential) is a crucial indicator for assessing the stability of colloidal dispersions, simultaneously influencing both physical and chemical adsorption processes. According to Stern’s double-layer theory, the electrochemical interface model is divided into two layers: the adsorption layer and the diffusion layer [28]. The interface where the adsorption layer (Stern layer) combines with a certain internal diffusion layer and undergoes relative movement with the dispersion medium is called the slip plane. The potential at which the particle surface potential drops to the slip plane is called the ζ potential. The polarity of a mineral’s zeta potential determines the charge of its surface. A positive zeta potential indicates a higher concentration of Ca^2+^ and Mg^2+^ ions, resulting in greater surface activity, stronger alkalinity, and enhanced adsorption affinity with asphalt molecules [21]. Jia [29] discovered that six single minerals exhibit surface charge properties: calcite carries a positive charge, while the other five carry negative charges. The absolute magnitude of the ζ potential follows this order: quartz > biotite > calcite > plagioclase > amphibole > pyroxene. Calcite was found to possess the strongest adhesion to asphalt, while quartz demonstrated the weakest.

Surface free energy comprises both a dispersion component and a polar component, wherein the dispersion component is also termed the van der Waals force, while the polar component is known as the Lewis acid–base interaction. The polar component of mineral aggregates significantly influences asphalt–aggregate adhesion. Cheng’s research [30] confirmed that aggregates with higher polar components exhibit poorer adhesion to asphalt. Materials with greater polarity possess stronger adsorption capacity for water molecules, rendering such aggregates more prone to adhesion failure with asphalt. This failure leads to aggregate detachment and subsequent pavement deterioration. Furthermore, analysis of the electrical potentials between different asphalt constituents and aggregate components revealed that asphaltic acids and asphaltic acid anhydrides are anionic asphalt constituents bearing negative charges. Consequently, positively charged limestone exhibits stronger adsorption interactions with asphalt than negatively charged granite, resulting in superior adhesion performance.

### 2.2. Chemical Composition of Asphalt

Asphalt, as a binder, possesses excellent adhesive properties. The content and polarity of asphalt constituents are the primary factors influencing asphalt–aggregate adhesion. Du et al. [24] proposed that among the four asphalt fractions, asphalt contributes most significantly to interfacial adhesion. Zheng et al. [31] demonstrated, through molecular dynamics simulations and comparative analysis, that the saturated and aromatic fractions within asphalt exhibit favorable fluidity, forming smaller contact angles with mineral surfaces. This enhances the micro-adhesive forces at the asphalt–mineral aggregate interface. Following aging, some light fractions in the asphalt transform into macromolecular heavy fractions, reducing the flow rate of asphalt on mineral surfaces and diminishing the mineral adsorption of asphalt. This leads to diminished adhesive capacity between asphalt and aggregates. Using grey relational analysis, they concluded that aromatic and saturated content significantly influence adhesive properties, while the asphalt matrix has the least impact. Zhang et al. [32] analyzed the effects of the chloride environment on the chemical composition and microstructure of asphalt binders through four-component tests and AFM experiments. They observed substantial alterations in the chemical composition of asphalt binders under salt erosion, alongside varying degrees of reduction in microstructural characteristics, such as surface roughness and the area ratio of “honeycomb structures”. Wei et al. [33] examined performance alterations in bituminous binders under coupled ultraviolet radiation and salt exposure. During aging, light fractions migrated towards heavier fractions; salt presence intensified this migration, accelerating bituminous degradation. Hao et al. [34] compared the adhesion of three asphalt types from different oil sources with aggregates. They found that adhesion negatively correlated with the mass fractions of gum and asphaltene and positively correlated with the saturated and aromatic fractions. However, the correlations differed between asphalt types from various oil sources. Lin et al. [35] employed molecular dynamics simulations to evaluate the contribution of four components to asphalt adhesion, concluding that adhesive capacity is primarily provided by the gum and aromatic fractions.

**Figure 3:**
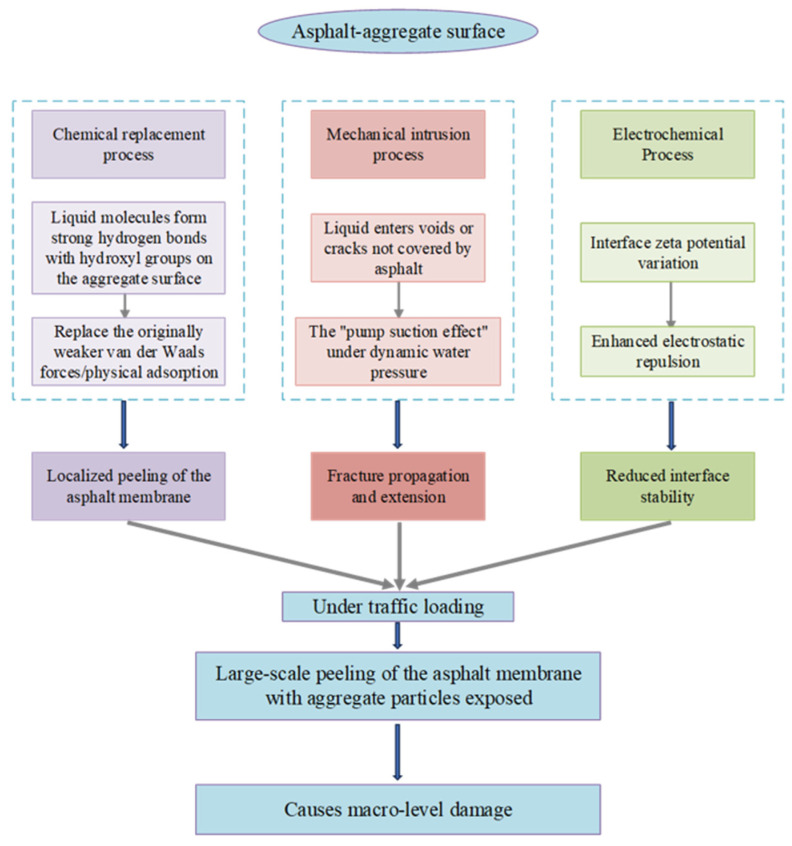
The schematic diagram of the degradation pathway.

Huang et al. [36] believed that the fundamental reason why asphalt and aggregate can bond is that both have polarity. The asphaltene and resin molecules in the asphalt component present an asymmetric structure and contain several active functional groups, forming the polar component of asphalt. When they are close to the surface of the polar aggregate, they can better complete the asphalt–aggregate bond. When these approach the polar aggregate surface, effective asphalt–aggregate bonding can be achieved. Yu et al. [37] employed molecular simulation techniques to investigate the interfacial adhesion mechanism between SBS asphalt and various aggregates under ultraviolet aging conditions. Analyzing the contribution of each SBS asphalt component to adhesion energy, they concluded that the increased polar functional groups formed during SBS asphalt aging influence interfacial adhesion work. Luo [38] posited that the limited polar content within asphalt facilitates enhanced physical bonding with the dispersed phase of alkaline aggregates, thereby improving interfacial stability. The interaction between asphalt and aggregate is the fundamental factor determining their adhesion. It is not a single mechanism but a complex coupling of physical, chemical, and mechanical effects. These interactions directly determine the mixture’s strength, durability, and resistance to water damage. A schematic diagram of the degradation pathway is shown in Figure 3.

### 2.3. External Environmental Influences

Asphalt pavements, when subjected to prolonged exposure in the natural environment, undergo phenomena, such as freeze–thaw cycles, wet–dry cycles, and vehicular loading, thereby coming into contact with moisture, salts, and other substances. Over time, this leads to the development of defects, such as cracking and spalling, the fundamental cause of which lies in the degradation of the asphalt–aggregate bond [39,40]. Consequently, numerous researchers have investigated the impact of external factors on this asphalt–aggregate adhesion.

Fakhr et al. [41] analyzed the effects of different chloride salts (NaCl, CaCl_2_, and MgCl_2_) on the moisture sensitivity of warm-mix asphalt mixtures. Their results indicated that the corrosive damage to asphalt mixtures caused by these salts occurred in the following order: MgCl_2_ > NaCl > CaCl_2_. Liu et al. [42] investigated the interfacial properties of asphalt–aggregate systems under humid conditions, discovering that moisture intrusion causes spontaneous separation between asphalt and aggregate surfaces. This occurs because aggregate particles readily adsorb water molecules; the accumulation of water molecules displaces asphalt molecules, ultimately leading to adhesion failure. Fauzia et al. [43] examined combined water–tire–pavement interactions, finding that moisture presence in asphalt mixtures accelerated surface cracking and rutting formation, with faster and more severe adhesion failure between asphalt and aggregates. Qian et al. [44] believed that the coupling of different water environments (salt, acid) and UV accelerated the aging of asphalt, resulting in a decrease in the saturated, aromatic, and resin contents, an increase in the asphalt content, and an enhancement in carbonyl and sub-broken group absorption peaks. The results also showed that under the same UV intensity, the influence of short-wavelength UV on asphalt aging was more serious.

Jin [45] found that the influence of environmental coupling conditions on asphalt aging is light acid > light water > light, light salt > light water > light. At the same time, the comparison of carbonyl and sulfoxide indices at the micro level also follows this order. The coupling of light, acid, and salt causes the most serious damage to the asphalt structure. The addition of a warm mix agent can reduce the mixing temperature of the asphalt mixture by 10–50 °C, which has a significant effect on reducing energy consumption and greenhouse gas emissions [46]. Sun et al. [47] observed that the addition of various warm-mix additives reduces adhesion. However, in water-saturated conditions, the adhesion system of warm-mix asphalt (incorporating Sasobit-type warm-mix additive H01 and surfactant-type warm-mix additive H02) with granite is more susceptible to damage than that with basalt, contrary to the behavior observed in dry conditions. Yang [48] thought that the damage caused by the sulfate, humidity, temperature, and ultraviolet radiation coupling effect on the performance of asphalt and a mixture was far more than the single-factor superposition effect, indicating that multi-physical field coupling is the core mechanism to accelerate a deterioration in material performance. Chen [49] observed that thermal-oxidative and salt-erosion effects reduce asphalt’s surface free energy. As asphalt ages, its contact angle with distilled water decreases, indicating increased hydrophilicity and heightened susceptibility to water damage. Du [50] subjected three asphalt types to dry–wet and freeze–thaw cycles using simulated seawater. Comparing adhesion properties before and after cycles via contact angle, FT-IR, and AFM tests revealed reduced adhesion work for all three after salt erosion cycles. When contrasted with asphalt adhesion work after equivalent cycles under pure water erosion, the addition of salt was concluded to accelerate the degradation of asphalt–aggregate adhesion. As freeze–thaw cycles progress, repeated freezing and thawing not only cause immersion corrosion by salt solutions but also generate frost heave forces within the asphalt–aggregate matrix due to ice expansion, accelerating the deterioration in adhesion properties [51,52]. Chu et al. [53] compared the performance of asphalt mixtures subjected to repeated sulfate and chloride freeze–thaw cycles, concluding that sulfates exert a greater impact on the water stability of asphalt mixtures than chlorides. This is attributed to the tendency of sodium sulfate crystals to agglomerate during crystallization, and the expansion of sodium sulfate crystals (the volume of the crystalline product increases to 3.1 times its original size) causes greater damage to the asphalt mixture. Yu et al. [54] believed that salt solutions undergo salt crystallization through freeze–thaw cycles, and the resulting crystallization pressure can compromise the structural stability of asphalt mixtures. Furthermore, crystallization pressure is positively correlated with the concentration of the salt solution. In 1939, scholars [55] first derived an equation relating the maximum stress (p) generated by solid-phase crystallization to the supersaturation of the solution, providing a theoretical basis for quantifying the impact of salt crystallization on the asphalt–aggregate interface. However, this theory has rarely been utilized for analysis. Fan [56] subjected asphalt to aging using different salt types, concluding that chloride, nitrate, and sulphate salts all react chemically with asphalt to generate new functional groups, thereby affecting asphalt adhesion. It was further determined that chloride salts influence damage depth, while nitrate and sulphate salts affect damage severity.

## 3. Evaluation Methods for Asphalt–Aggregate Bonding

### 3.1. Laboratory Test Methods

The adhesion failure between asphalt and aggregate is a typical progressive process triggered by micro-interface failure, ultimately leading to macro-scale pavement deterioration. Initial damage occurs due to physical/chemical changes at the nanometer (nm) or even micrometer (μm) scale, forming micro-cracks or voids at the interface and reducing adhesion strength. Over time, as these effects accumulate, the micro-cracks and voids begin to connect, forming visible micro-cracks. Ultimately, this leads to macroscopic (m) pavement damage visible to the naked eye. In studies of asphalt–aggregate adhesion, traditional macroscopic testing methods have long been widely employed in practical engineering assessments and water stability analyses due to their operational simplicity and intuitive results. These methods primarily include the boiling water test, water immersion test, contact angle test, and pull-off test. The boiling water test enables a rapid qualitative assessment by observing the state of asphalt film peeling from the aggregate surface after boiling. The pull-off test quantitatively measures the tensile bond strength at the asphalt–aggregate interface, directly reflecting the mechanical indicator of adhesion performance. It is currently one of the widely adopted standardized evaluation methods. However, traditional macroscopic testing methods are predominantly qualitative analyses, with experimental results significantly influenced by subjective personal judgment, making it challenging to achieve accurate quantitative characterization of indicators.

**Figure 4:**
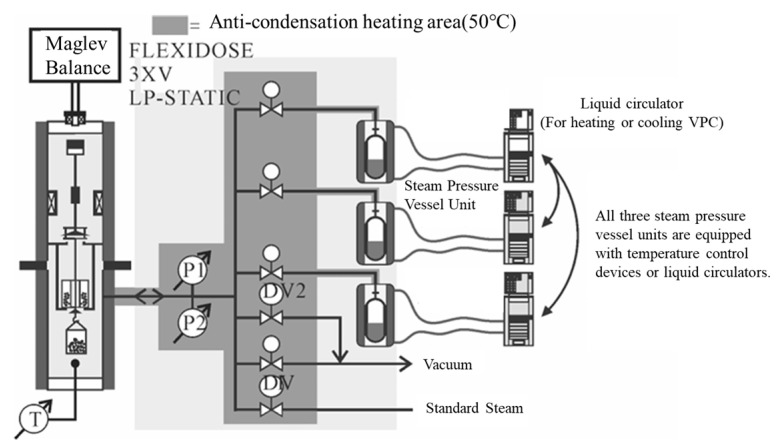
Maglev weight balance system [57].

In response to the limitations of traditional macro methods, some scholars have developed new experimental methods and combined innovative experimental instruments to achieve more diverse and effective environmental simulation and indicator testing. The Rolling Bottle Test (RBT) involves placing aggregate particles wrapped in asphalt into a water-filled glass bottle and rotating it to simulate the collision between dynamic water erosion and aggregate particles, which better simulates the external effects on the asphalt–aggregate mixture interface in actual working conditions. MIST simulates the scenario of asphalt pavement subjected to vehicle loads under wet conditions by continuously injecting and extracting water into an asphalt mixture specimen and applying a certain load to the mixture specimen at a specified temperature. To assess the surface energy of aggregate particles, the Luo Rong research group at Wuhan University of Technology [57] customized a magnetic levitation weight balance system (as shown in Figure 4). This system maintains constant vapor pressure within the sample chamber. By calculating the vapor adsorption on the aggregate surface, parameters such as specific surface area and diffusion pressure can be determined, ultimately yielding the surface energy of aggregate particles. This method provides greater accuracy compared to calculating solid surface energy via contact angle measurements. The Zhang Jizhe research group at Shandong University [58] independently developed a micromechanical testing system for the asphalt–aggregate interface and a pressure device capable of simulating pavement pore water pressure. The Chu Ci research group at Chang’an University [59] independently developed an asphalt mixture salt erosion dynamic water scouring apparatus. The Wu Jiantao research group at Hohai University [60] independently developed a “water aging”-coupled environmental simulation apparatus. Details of these apparatuses are presented in Table 2.

Asphalt should have appropriate rheological properties (such as easy-to-wrap aggregate at construction temperature and resistance to deformation at service temperature) to achieve and maintain good adhesion. At the same time, the failure of adhesion (such as aggregate peeling) often shows the specific rheological behavior of an asphalt membrane under the action of load and water (such as sliding, thinning, and fracture). Therefore, some scholars use the test results of asphalt rheological properties to indirectly evaluate the adhesion characteristics of the asphalt–aggregate interface.

When the asphalt–aggregate interface is subjected to external effects, many changes will occur at the micro level, such as the competitive adsorption of water molecules and asphalt on the aggregate surface, the change in new functional groups and surface morphology caused by asphalt aging, etc. These micro changes are very helpful in understanding the failure mechanism of asphalt–aggregate interface adhesion and are crucial to the development of anti-stripping agents, the selection of modified asphalt, and the optimization of aggregate surface properties. Modern material characterization techniques, such as atomic force microscopy (AFM), environmental scanning electron microscopy (ESEM), X-ray photoelectron spectroscopy (XPS), and CT, are powerful tools for direct observation and quantitative analysis of these microstructure changes.

Numerous scholars have employed diverse testing methods to determine various characterization parameters, investigating the impact of environmental changes on asphalt–aggregate adhesion. Key conclusions are summarized in Table 3.

The tensile strength at the asphalt–aggregate interface is a commonly used macro-level indicator for characterizing asphalt–aggregate adhesion performance. After exposure to different salt solutions, the reduction in pull-off strength can exceed 30%. The initial rate of decline is relatively rapid, subsequently stabilizing. Each salt solution exerts a different degree of influence on the tensile strength of specimens. At equivalent salt concentrations, specimens subjected to sulfate corrosion exhibit a greater reduction in tensile strength, and lower-concentration solutions exert a more pronounced effect on tensile strength. Table 4 presents variations in asphalt–aggregate interface tensile strength obtained by various researchers under different testing conditions.

The ER_1_ index, based on surface energy theory (which comprehensively considers the adhesion work between asphalt and aggregate, as well as the influence of the peeling work at the asphalt–aggregate–water interface), and the ER_2_ index (which further takes into account the cohesive work of asphalt mastic based on ER_1_) are commonly used characterization indicators at the microscopic level. As the number of salt cycles increases, both ER_1_ and ER_2_ exhibit a decreasing trend. Comparisons among different types of asphalt reveal that SBS-modified asphalt demonstrates improved adhesion properties compared to base asphalt, but its performance degradation becomes more pronounced with repeated salt corrosion cycles. Analysis of ER value changes across different aggregates indicates that limestone (carbonate mineral) exhibits superior performance compared to basalt (silicate mineral). Table 5 and Table 6 illustrate the changes in the asphalt–aggregate interface ER_1_ and ER_2_ indices under different testing conditions.

### 3.2. Molecular Dynamics Simulation

Molecular dynamics simulation (MD) has emerged as a significant computational tool in recent years, finding application and development within research on asphalt–aggregate adhesion. This methodology operates at the atomic and molecular scales. By constructing atomic models of asphalt components, aggregate crystals, and the interfacial system, it employs force fields such as COMPASS and COMPASSII to describe particle interactions. Utilizing simulation platforms like LAMMPS, it solves Newton’s equations of motion under specific temperature and pressure conditions, thereby achieving dynamic simulations of system evolution. By analyzing key parameters such as adhesion work, concentration distribution, interfacial energy, intermolecular forces (van der Waals forces, Coulombic forces, etc.), and molecular motion behavior (e.g., mean square displacement), it can profoundly reveal the micro-mechanisms and failure pathways at the asphalt–aggregate interface under conditions involving salt ions, moisture, and aging. Molecular dynamics simulations not only compensate for the limitations of macroscopic experiments in elucidating micro-mechanisms but also enable precise control and systematic analysis of multiple environmental variables (humidity, salt type, ion concentration, etc.). This provides a powerful theoretical tool for understanding asphalt–aggregate interface behavior under a complex salt corrosion environment [71,72].

**Figure 5:**
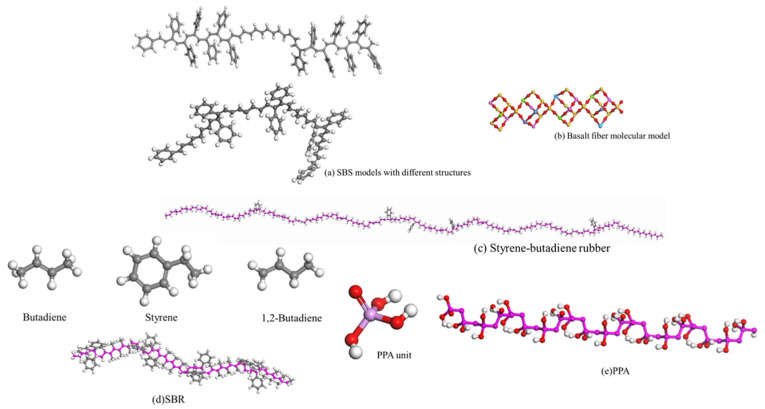
Molecular models of different modifiers.

**Figure 6:**
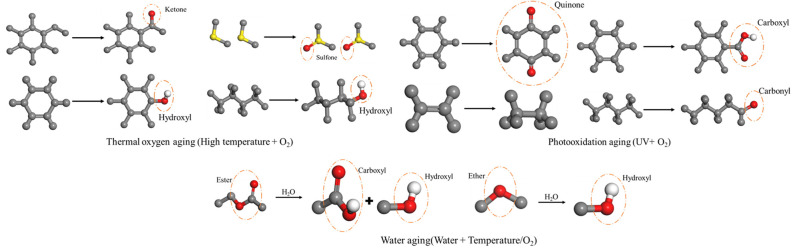
Schematic of the aging principle.

**Figure 7:**
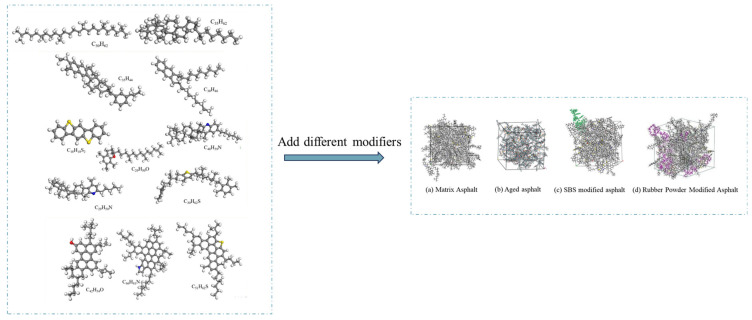
Different asphalt molecular models.

**Figure 8:**
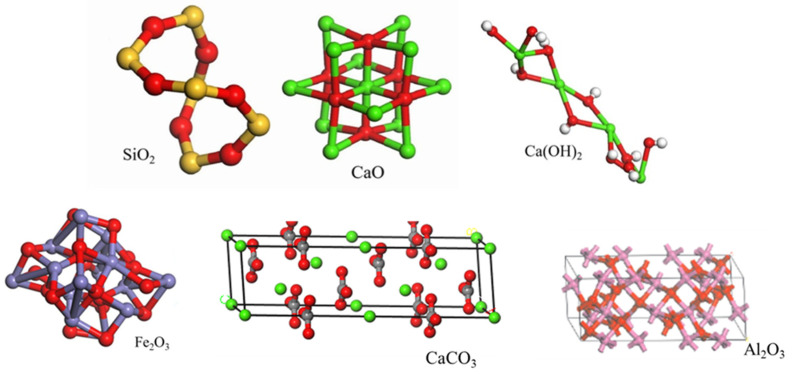
Aggregate model.

**Figure 9:**
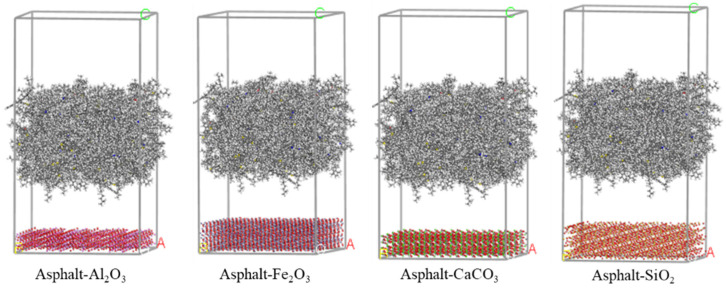
Asphalt–aggregate interface model. (A, C as the coordinate axis).

The asphalt molecular model predominantly employed in simulations is the twelve-component model proposed by Derek D. Li [73]. To simulate the properties of modified and aged asphalt, researchers have developed distinct models for asphalt modifiers and aged asphalt. Selected modifier molecular models are illustrated in Figure 5 [74]. Following aging, the structural composition of asphalt changes, generating new functional groups. The aging mechanism is schematically illustrated in Figure 6. Asphalt molecules are constructed by incorporating asphalt component models and modifier molecular models in specific proportions, as schematically depicted in Figure 7. Aggregate molecules are predominantly modeled using representative oxide crystals, with common oxide models depicted in Figure 8 [75]. As computational simulation techniques advance, numerous scholars employ molecular dynamics methods to construct diverse asphalt–aggregate models, as shown in Figure 9. They calculate characteristic parameters under varying conditions to investigate the damage mechanisms affecting asphalt–aggregate adhesion, with key findings summarized in Table 7.

### 3.3. Aggregate–Asphalt Bonding Performance Prediction

Given the complex mechanisms underlying the influence of salt-enriched environments on asphalt–aggregate adhesion, involving multifactorial and nonlinear interactions, traditional experimental and theoretical analyses alone are insufficient to comprehensively predict its long-term performance evolution. Therefore, incorporating machine learning methods to develop predictive models facilitates the integration of multi-source data, identification of underlying patterns, and quantitative assessment and trend forecasting of adhesion behavior under varying salt conditions. This approach provides more scientific and efficient decision support for engineering practice. Machine learning is a complex interdisciplinary field encompassing computer science, statistics, mathematics, and engineering. Its essence relies upon vast datasets, employing specific algorithmic rules to enable computers to autonomously simulate human learning processes. Through continuous data learning, it enhances relevant performance and makes intelligent judgments [87,88,89]. Machine learning modeling can be segmented into three phases: data storage, input, and pre-processing; model construction and iterative optimization during learning; and final output of test data results. Presently, machine learning algorithms have been applied within road engineering research, encompassing pavement defect analysis and prediction, asphalt mixtures, and asphalt materials [90,91,92,93].

Hamedi et al. [94] compared the use of Genetic Expression Programming (GEP) and Multi-Genetic Genetic Programming (MGGP) to develop predictive models for the water stability of asphalt mixtures, forecasting two performance indicators: the Indentation Stress Point (ISP) and the Stress Slope (SS). For ISP prediction, the R^2^ values for the MGGP and GEP models were 0.981 and 0.956, respectively; for SS prediction, the R^2^ values were 0.974 and 0.928, respectively. The MGGP model was selected as the superior model based on error metrics and thus employed for subsequent estimation. Babagoli et al. [95] employed a multi-layer perceptron neural network (MLPNN) within an artificial neural network (ANN) alongside a support vector regression (SVR) algorithm to model and predict the effects of various modifiers on the water stability of asphalt mixtures. The predictive model results indicated that both the SVR and ANN models demonstrated good accuracy in estimating the fracture energy ratio (FER), resilience modulus ratio (RMR), and tensile strength ratio (TSR). Moreover, the ANN model outperformed the SVR model in training, testing, and overall data performance across all scenarios. Low-temperature cracking in asphalt mixtures is fundamentally related to two factors: the cohesive strength within the asphalt binder and the adhesive strength at the asphalt–aggregate interface. Therefore, the quality of asphalt–aggregate adhesion can be indirectly assessed through low-temperature performance testing. Wu et al. [96] constructed a particle swarm optimization (PSO)-optimized backpropagation (BP) neural network to predict the low-temperature performance of polyester fiber-reinforced asphalt mixtures under various dry–wet cycling conditions. As a global intelligent search algorithm, PSO iteratively explores the search space to obtain optimal solutions. The PSO-BP neural network demonstrated enhanced capability in handling nonlinear data relationships with increasing structural complexity. Validation through error coefficients confirmed the PSO-BP model’s superior predictive performance relative to the BP model. Arifuzzaman et al. [97] employed a predictive modeling and machine learning technique known as Classification and Regression Trees (CARTs) to forecast the adhesion properties of oxidized modified asphalt. Prediction results were obtained, with CART analysis outcomes compared against regression model outputs. CARTs analyzed more specific relationships between different variables, accounting for the real-field oxidation and chemical properties of asphalt at the nano-scale. It was concluded that this model can accurately predict the adhesion properties of modified asphalt. Lü [98] employed machine learning algorithms to construct an asphalt fatigue life prediction model using thixotropic-dominated domain characteristic parameters as inputs. This model can predict the fatigue life of various asphalt types under diverse test conditions, with its accuracy validated. Sun [89] employed a particle swarm optimization (PSO)-enhanced support vector machine (SVM) to establish a fatigue life prediction model for SMC/SBSCMA mixtures. By comparing outputs from the M5’s decision tree, artificial neural network (ANN), and PSO-optimized SVM models, the study concluded that PSO-SVM delivers superior fatigue life prediction performance for these mixtures. Meng [99] built a Weibull damage model to explore the damage changes in an asphalt mixture under the action of multi-factor salt erosion. The reliability of the model was verified by actual tests. The correlation coefficient between the predicted value and the measured value of the model was higher than 0.98. It was considered that the damage accumulation law under the action of the dynamic water salt erosion photothermal cycle could be accurately characterized by parameter iteration optimization, which provided a quantitative analysis tool for the performance damage of the asphalt–aggregate interface and the durability evaluation of the mixture. Liu [100] established the GM (1, n) prediction model of grey theory to predict the salt frost resistance of asphalt–aggregate. The relative error between the predicted value of the model and the measured value in the laboratory was 1.33%. It was determined that the model could better predict the future development.

## 4. Improvement Measures

Enhancing the adhesion between asphalt and aggregate can effectively mitigate the current road defects caused by water damage to road materials. Based on research and analysis of adhesion performance, the primary methods for improving adhesion currently include the addition of asphalt modifiers and aggregate modification treatments.

### 4.1. Asphalt Modification

**Figure 10:**
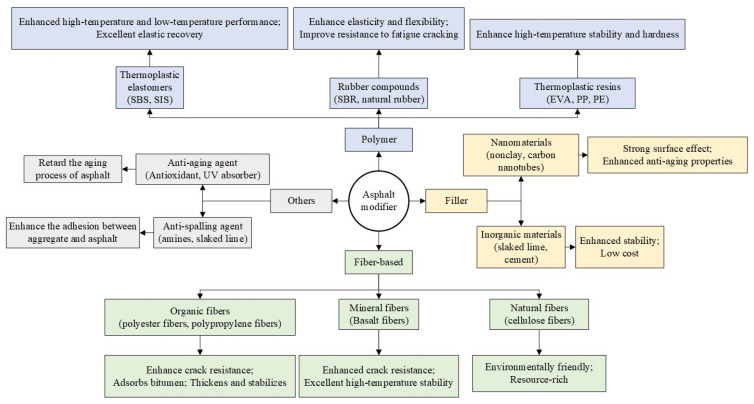
Classification and advantages of asphalt modifiers.

Asphalt, as a binder in asphalt mixtures, plays a crucial role in the stability of mixtures. The performance of asphalt itself contributes greatly to the adhesion effect of the asphalt–aggregate interface. By modifying it, the adhesion performance of asphalt itself can be improved, which has a positive effect on the adhesion of the asphalt–aggregate interface. Meanwhile, practical engineering demands for asphalt performance have increased. Consequently, numerous scholars have investigated the efficacy of various substances as asphalt modifiers. Common asphalt modifier classifications and their advantages are illustrated in Figure 10.

Water-based polyurethane, utilizing water as a solvent, offers advantages such as safety, reliability, and excellent compatibility. It finds extensive application across industries, including light textiles, coatings, building materials, and adhesives. Wang [101] observed that 4% water-based polyurethane emulsified asphalt exhibited no aggregate stripping during water-boiling tests. Aggregate surfaces were uniformly coated with an asphalt film, while asphalt particles within the emulsion formed an interlinked network structure with the water-based polyurethane emulsion material, thereby enhancing the emulsified asphalt’s adhesion.

Polyphosphoric acid (PPA), characterized by its excellent stability and high viscosity, is increasingly employed in highway engineering as an economical chemical modifier. Wang et al. [102] prepared PPA/SBS-modified asphalt, where PPA increased the effective contact area between aggregates and SBS-modified asphalt, thereby enhancing the adhesion of the composite modified asphalt. The adhesion of the composite-modified asphalt was maximized at a PPA dosage of 0.5%. Zhou [103] determined, through frequency scanning, that PPA composite modified asphalt exhibited the strongest interaction capacity with aggregates. Applying the time–temperature equivalence principle, it was found that at elevated temperatures, PPA composite modified asphalt demonstrated relatively superior wetting effects with limestone aggregates, indicating enhanced adhesion performance. Liu et al. [104] experimentally demonstrated that incorporating polyphosphoric acid into rubber-modified asphalt promotes gelation, enhancing storage stability, mitigating rubber powder segregation, and improving both high-temperature stability and aggregate adhesion. Guo [105] observed that adding PPA under aqueous conditions disrupted adhesion at the asphalt–aggregate interface. However, PPA-modified asphalt reduced stripping loss and enhanced asphalt–aggregate bonding. Notably, PPA/SBS and PPA/SBR composite modified asphalts exhibited nearly identical improvement levels. Li [106] modified asphalt by blending varying proportions of PPA and sugarcane fiber (SF). When incorporating 1.0% PPA + 3% SF, the ER value increased from the original asphalt’s 0.69 to 1.06 after salt erosion testing, demonstrating a significant improvement in adhesion performance. In a large number of current studies, there is still considerable controversy regarding the optimal dosage of PPA and the best combination for composite modification. For the same composite modification combination, there is still no consensus on the optimal proportion of each modifier.

As one of the most promising contemporary materials, nanomaterials have found extensive application across diverse fields, with numerous scholars employing them in asphalt modification [107]. However, the specific types of nanomaterials and the proportions in which different nanomaterials are added vary, and the optimal mixing ratio is still under consideration. Wang et al. [108] compared the adhesion changes in nanomaterial-modified asphalt across different dimensional scales. From zero-dimensional to two-dimensional nanomaterials (nano-CaCO_3_, multi-walled carbon nanotubes, nano-montmorillonite), all reduced the polar component of asphalt to varying degrees while increasing the dispersion component. This enhanced surface energy-related parameters, thereby improving adhesion between the asphalt and aggregate. Zhang [109] employed composite modification of SBS asphalt using nano-TiO_2_ and OMMT (organic nano-montmorillonite). Post-modification, all performance metrics of the asphalt surpassed those of the SBS asphalt. Moreover, following both short-term and long-term aging, the composite-modified asphalt retained superior adhesion properties compared to the SBS-modified asphalt. Xiao [110] observed that n-ZnO and n-TiO_2_ improve asphalt adhesion, exhibiting superior bonding compared to SBS-modified asphalt. Dispersion tests revealed excellent stability in n-ZnO and n-TiO_2_-modified asphalts, with n-ZnO/SBS and n-TiO_2_/SBS demonstrating enhanced storage stability over SBS-modified asphalt. Guo et al. [111] utilized nano-SiO_2_ to modify asphalt, observing simultaneous physical and chemical interactions between nanoparticles and asphalt. This enhanced stability, improved physical interlocking between asphalt and aggregates, and elevated the comprehensive performance of the asphalt.

Basalt fibers exhibit high strength and biodegradability, alongside resistance to chemical corrosion and high temperatures, rendering them extensively employed in road engineering. Lou [112] investigated changes in asphalt spectrum characteristics before and after basalt fiber incorporation via FTIR analysis. It was concluded that no chemical reaction producing new characteristic peaks occurred upon fiber addition, suggesting that interfacial interactions are primarily governed by physical adsorption. Cheng [113] applied phthalic acid ester coupling agents to modify conventional basalt fibers, producing modified asphalt. The modified basalt fibers exhibited improved mechanical properties, increased surface roughness, and a 20.5% increase in adhesion work compared to the unmodified fibers. Concurrently, the enhanced interfacial adhesion and interaction between the modified fibers and asphalt further strengthened the adhesion between asphalt and aggregates.

Baldino et al. [114] incorporated three distinct surfactants into asphalt: cationic surfactant modifiers (comprising quaternary ammonium and halide ions), organ silane surfactants, and primary alkylamine surfactants. They analyzed their effects on the salt erosion resistance of asphalt mixtures. Their results indicated that organ silane surfactants effectively enhance the interfacial adhesion between asphalt binders and aggregates under salt erosion conditions. Dan et al. [115] utilized organic the anti-stripping agents AMRIII, LQ-2020, and ultrafine hydrated lime (HL) to investigate the effects of anti-stripping agents on asphalt and asphalt–aggregate interfaces. Their results indicated that incorporating anti-stripping agents enhances asphalt mechanical strength while strengthening cohesion and adhesion between asphalt and aggregates, thereby improving the water stability of asphalt mixtures.

### 4.2. Aggregate Modification

**Figure 11:**
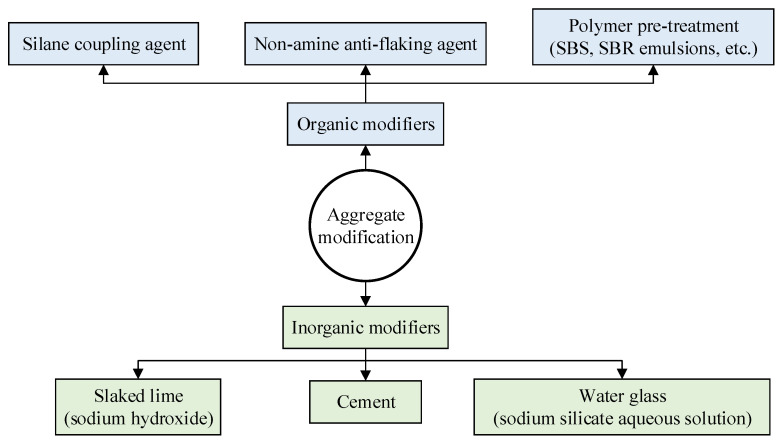
Classification of aggregate modifiers.

Alkaline aggregates provide superior adhesion to asphalt in asphalt mixtures, whereas acidic aggregates exhibit poor bonding properties with asphalt, limiting their application in asphalt pavement engineering. Commonly used aggregate modifiers are categorized as shown in Figure 11.

Xuan et al. [116] developed an aggregate modifier utilizing anhydrous ethanol as the solvent, with solute components comprising silane coupling agents, epoxy resin, and curing agents. Following atomization and spraying onto heated aggregate surfaces, solvent evaporation leaves the active solutes to form a dense encapsulating film on the aggregate surface, thereby enhancing adhesion between acidic aggregate particles and asphalt. Different types of silane coupling agents have varying effects on the modification of aggregates. Wang et al. [117] utilized the KH-550 silane coupling agent to modify crushed stone surfaces. The modified surfaces exhibited methylene groups that enhanced the bonding properties between the aggregate and asphalt. At a coupling agent dosage of 0.6%, the low-temperature adhesion between the asphalt and crushed stone aggregate was found to be strongest, with stone loss reduced by 50.7%. Following the MIST test, the residual splitting strength ratio increased by 7.9%. Peng [118] treated aggregates with varying proportions of silane coupling agents, observing that these agents significantly improved asphalt–aggregate bonding. Li et al. [119] found that increasing the modifier dosage markedly enhanced adhesion between acidic aggregates and asphalt, though excessive amounts exhibited a trend of initially improved, then deteriorated adhesion. Ding et al. [120] investigated the modification effect of the silane coupling agent KH-560 on granite aggregates. KH-560 significantly enhanced the adhesion between acidic aggregates and asphalt, improving the mixture’s water stability and low-temperature performance. Concurrently, the addition of silane coupling agents markedly increased the concentration distribution of asphalt molecules on the aggregate surface. Yang et al. [121] investigated the effects of the silane coupling agent KH-792 on the surface properties of acidic aggregates and the performance of asphalt mixtures. Their results indicated that the silane coupling agent was chemically grafted onto the aggregate surface, leading to a marked increase in the contact angle and a significant reduction in hydrophilicity. This effectively improved the adhesion between the asphalt and aggregates, thereby enhancing the water stability of the mixture.

**Figure 12:**
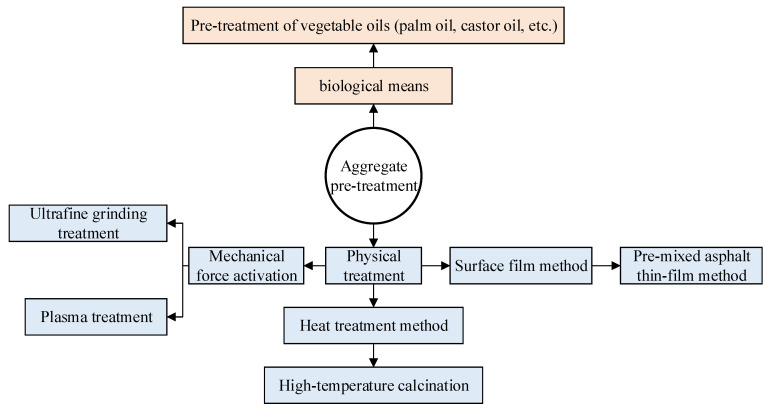
Aggregate pre-treatment methods.

**Figure 13:**
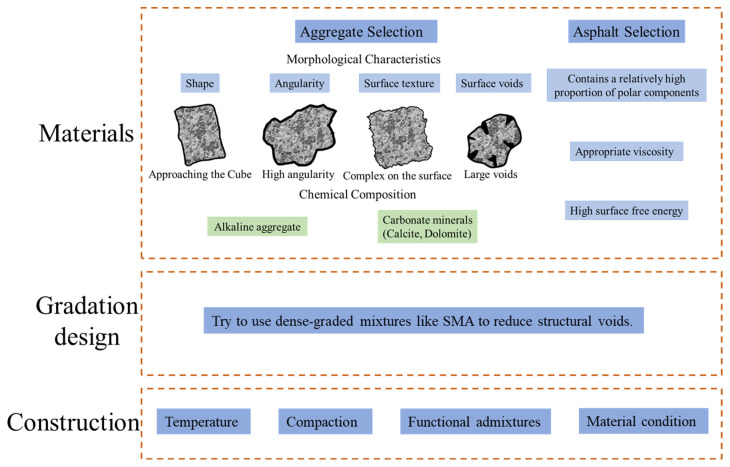
The considerations from material selection to construction.

Zhu [122] modified aggregate surfaces using calcium hydroxide, iron oxide, and aluminum oxide powders. All three modifiers enhanced adhesion between aggregates and asphalt. A comparative analysis of surface modification versus external admixture methods for improving asphalt–aggregate adhesion concluded that surface modification yielded superior results when employing identical modifiers. Beyond direct surface modification of aggregate particles, pre-treatment methods can also enhance adhesion between aggregates and asphalt. The classification of pre-treatment methods is shown in Figure 12. Beyond these modification measures, reducing voids within the asphalt mixture can be achieved by selecting a skeleton-dense aggregate gradation, optimizing the asphalt mixture design, and appropriately increasing construction compaction. This approach minimizes pathways for external environmental factors, such as salt and moisture, to penetrate the internal structure, thereby reducing damage to the asphalt–aggregate interface adhesion and enhancing the mixture’s structural stability and durability. The considerations from material selection to construction are shown in Figure 13.

## 5. Conclusions

Salt-enriched environments exert a pronounced detrimental effect on the adhesion at the asphalt–aggregate interface, with degradation mechanisms being complex and multifaceted. These involve the synergistic interaction of multiple physicochemical processes, including salt crystallization pressure, water ingress, and ion exchange. This paper systematically reviews research progress on asphalt–aggregate adhesion in such environments, elucidating the mechanisms of adhesion degradation through intrinsic factors (aggregate chemical composition and surface morphology; asphalt components and polarity) and extrinsic factors (moisture, salts, temperature, aging, loading). Research indicates that alkaline constituents, such as CaO in aggregates, enhance adhesion, whereas acidic components, like SiO_2_, readily induce interfacial delamination. Light fractions (saturated and aromatic components) in asphalt favor initial adhesion, but increased heavy fractions post-aging diminish their diffusion and adsorption capabilities. Within the external environment, chloride and sulphate salts exhibit differing degrees of corrosive impact, with MgCl_2_ proving more destructive than NaCl and CaCl_2_. Furthermore, the coupled effects of moisture, temperature, and loading significantly accelerate interfacial failure.

Regarding evaluation methods, traditional macroscopic tests, such as water boiling and pull-off tests, while operationally straightforward, struggle to elucidate microstructural mechanisms. Molecular dynamics simulations, however, reveal the competitive adsorption behavior and diffusion patterns of water molecules and salt ions at the interface at the atomic scale, effectively complementing the limitations of macroscopic research. Regarding improvement measures, asphalt modification (e.g., polyurethane, polyphosphate, nanomaterials) and aggregate surface treatment (e.g., silane coupling agents) have been proven to significantly enhance interfacial adhesion and salt resistance, with notable progress achieved particularly in modifying acidic aggregates. Current applications of machine learning in asphalt–aggregate adhesion research demonstrate promising predictive capabilities and modeling potential. Existing studies have successfully employed machine learning to predict key indicators of asphalt mixtures, including water stability, adhesion performance, and fatigue life. These models demonstrate excellent fitting accuracy and generalization capabilities, particularly in addressing the complex degradation of adhesion involving multi-factor coupling and nonlinear relationships—an advantage not possessed by traditional empirical methods.

The current research has preliminarily established a multi-scale evaluation system and modification technology pathway for asphalt–aggregate adhesion under salt corrosion environments, providing theoretical foundations for asphalt pavement design and maintenance in salt-rich regions. However, the existing research predominantly focuses on single factors or short-term aging effects, with insufficient understanding of multi-field coupling effects, long-term performance evolution, and practical engineering applicability. Consequently, research on asphalt–aggregate adhesion in salt-enriched environments currently faces the following challenges:

(1) Quantification of Accelerated Aging and Failure Mechanisms in Multi-factor Coupling: A multi-factor coupled accelerated aging test platform should be established to quantify the contribution of each environmental factor to adhesion degradation and reveal the evolution patterns of interfacial damage under the synergistic effects of dynamic loading and salt corrosion. Considerations should include precisely controlling environmental simulation conditions; isolating and quantifying the independent effects of individual components from synergistic interactions; constructing damage allocation models; and establishing mechanical evolution models to determine the relationship between interface changes and salt concentration, as well as applied stress.

(2) Intelligent Monitoring and Data-Driven Failure Prediction: By integrating sensing technology, the Internet of Things, and artificial intelligence, real-time monitoring and degradation prediction of interface conditions are achieved, providing data support for preventive maintenance. Future research should consider how to achieve synchronous acquisition of all parameters; utilize deep learning models (such as LSTM, Transformer, etc.) to construct dynamic prediction models; precisely define “interface failure”; and build early warning models.

(3) Quantification of Composite Salt Erosion Mechanisms and Synergistic Effects: Future research should elucidate the synergistic or antagonistic effects of multiple salts in composite salt systems and establish a composite salt–multi-factor coupling experimental system that closely mimics real-world environments; consider the influence mechanisms of different ions on the electrochemical corrosion potential and ion migration rate at the asphalt–aggregate interface within composite salt solutions and whether a dominant ion exists; examine the synergistic effects of various salts in accelerating interfacial failure and determine how to quantify their synergistic coefficients; and investigate how this coefficient varies under different concentration ratios and how crystallization pressure generated during crystallization can be quantitatively characterized.

(4) Durability and Microstructural Mechanisms of Novel Functional Materials: Future research should systematically evaluate the performance of novel functional materials under long-term salt corrosion environments, elucidate their micro-mechanisms of reinforcement and self-healing, and develop composite modification strategies.

(5) Standard Establishment, Technical Validation, and Engineering Transformation: Future research should establish unified evaluation standards and technical guidelines to advance salt-resistant technologies from laboratory research to large-scale engineering applications; consider establishing a multi-scale, multi-indicator adhesion evaluation system encompassing macro-mechanics, microstructure, and chemical composition, while analyzing the correlation between each indicator and long-term service performance; and design a long-term performance tracking plan for physical engineering structures to validate the correspondence between accelerated indoor aging tests and actual field aging processes, thereby establishing an accelerated aging equivalence model.

(6) Interdisciplinary Mechanism Integration and Full Life Cycle Sustainability Assessment: Drawing upon theories of concrete reinforcement corrosion, future research should establish an ion migration–damage model applicable to asphalt pavement systems; simultaneously evaluate the environmental impact of novel materials and quantify the long-term benefits of salt-resistant technologies through life cycle analysis; and consider interdisciplinary analogies between ion transport and failure mechanisms, such as environmental leaching and ecological impact assessment of modifiers and life cycle analysis (LCA) of salt-resistant pavement versus conventional maintenance strategies.

## Figures and Tables

**Table 1:** The mineral composition and chemical constituents of various aggregates.

Common Aggregate Types	Principal Mineral Composition	Principal Chemical Composition
limestone	Calcite, dolomite, magnesite	CaCO_3_, MgO, Fe_2_O_3_
basalt	Pyrite, plagioclase, olivine	SiO_2_, Al_2_O_3_, Fe_2_O_3_
granite	Quartz, potassium feldspar, plagioclase feldspar	SiO_2_, Al_2_O_3_, K_2_O
dolomite	Quartz, feldspar, pyroxene	CaCO_3_, MgO, SiO_2_
dolerite	Pyroxene, plagioclase, olivine	SiO_2_, Al_2_O_3_, Fe_2_O_3_

**Table 2:** Information for different new-type devices.

Instrument Name	Schematic Diagram of the Instrument	Simulated Environment	Control Parameters	Characteristics	Source
Dynamic Water Pressure Simulation Apparatus	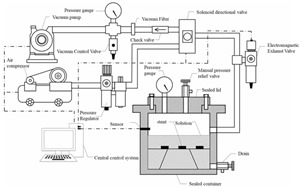	Dynamic water pressure	The magnitude of gas pressure within a sealed container and the duration of its application	Realistic simulation of asphalt–aggregate structures subjected to dynamic hydrostatic pressure erosion, enabling rapid alternating positive and negative pressure cycles for enhanced realism.	Zhang Jizhe, Shandong University [58]
Asphalt Mixture Salt Erosion Dynamic Water Scouring Apparatus	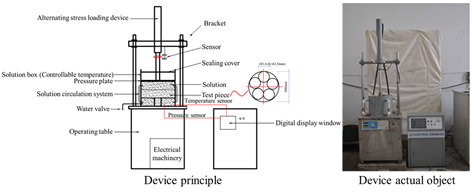	Erosion by flowing water	Temperature; pressure; number of flushes; flushing medium	Simulates the pumping action of pore water within asphalt mixtures under vehicular loads, thereby compensating for the limitations of static load testing.	Chu Ci, Chang’an University [59]
Simulation Apparatus for Coupled Environmental Effects of Water Aging	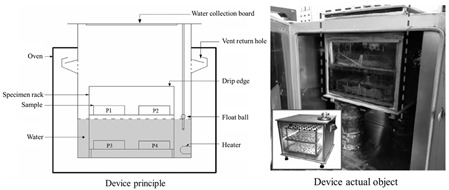	Aging–water damage coupling	Duration of the aging process; temperature; water exposure	Enables precise control of the drip volume, ensuring accurate regulation of the specimen’s water exposure.	Wu Jiantao, Hohai University [60]

**Table 7:** Model selection and main conclusions on asphalt–aggregate adhesion.

Asphalt Model	Aggregate Model	Force Field Selection	Indicator	Key Findings
Blend 20% CR and 3% SBS by mass ratio into AAA-1	CaCO_3_, 2CaO·SiO_2_, 3CaO·SiO_2_	COMPASS II	Adhesive work; radial relative concentration; mean displacement	Adhesion reduction for CaCO_3_, C_2_S, and C_3_S is 75%, 37%, and 8.9% in water and 86.5%, 66.8%, and 9.8% under salt corrosion, respectively. Salt corrosion enhances CR migration from the steel slag surface, weakening the asphalt–slag interaction and further reducing adhesion [76].
90# base asphalt, severely aged asphalt, mildly aged asphalt	SiO_2_	COMPASS	Fracture energy; interfacial bond strength	Salt solution permeation into the asphalt–aggregate interface markedly reduces bond strength and fracture resistance, posing a more severe threat than water penetration. The conditions governing pull-off, particularly load rate and temperature, play a pivotal role in controlling the mechanical response at the asphalt–aggregate interface [77].
SBS-modified asphalt; basalt fiber; KH550-modified basalt fiber	CaO, SiO_2_, Al_2_O_3_	COMPASS	Adhesion work; interfacial area; radial distribution function	The incorporation of basalt fibers enhances the adhesion between asphalt and aggregates. When modified with KH550, the adhesion strength increases by 16% compared to the unmodified state, facilitating improved bonding with aggregate oxides [78].
70# base asphalt; aged base asphalt; SBS-modified asphalt	SiO_2_, CaCO_3_	COMPASS	Adhesion work; peel work; mean displacement and interfacial energy	Alkaline aggregates adhere to asphalt through electrostatic forces, whereas acidic aggregates rely on van der Waals forces, resulting in poorer adhesion. SBS-modified asphalt exhibits optimal adhesion properties, with a significant reduction in adhesion between aged asphalt and aggregates observed [79].
Rubber-modified asphalt; matrix asphalt	CaO, SiO_2_, Al_2_O_3_, Fe_2_O_3_	COMPASS	Mean square displacement; interfacial energy; diffusion coefficient	Water diffusion at the rubber–asphalt–aggregate interface depends on temperature. Compared to SiO_2_ surfaces, CaO surfaces show a stronger ability to adsorb water. In saline environments, salts are the main cause of degradation. Higher salt concentrations in solutions enhance the diffusion of asphalt components, which weakens interfacial bonding [80].
Rubber-modified asphalt, matrix asphalt, SBS-modified asphalt	CaCO_3_	COMPASSII	Adhesion work; mean square displacement; radial relative concentrations; contact angle	Salt solutions are more readily adsorb and disperse on asphalt surfaces than aqueous solutions. This may lead to the dissolution of asphalt polar groups, causing asphalt film cracking and salt solution penetration into the asphalt interior. During salt solution migration, asphalt components redistribute, affecting adhesion performance [81].
Matrix asphalt	SiO_2_, CaCO_3_	COMPASSII	Diffusion coefficient; adhesion work; diffusion thickness; number of hydrogen bonds	Asphalt diffusion is minimal at the aggregate surface and increases with distance from it. Distribution depends on aggregate and solution properties, leading polar components to accumulate at the solution surface, while non-polar ones diffuse differently [82].
Twelve four-component asphalt molecular models; various de-icing agents	CaO, SiO_2_	COMPASSII	Interfacial adhesion energy; asphalt concentration on oxide surfaces; adhesion energy ratio	The ingress of a de-icing solution compromises the equilibrium stability of asphalt. Furthermore, once the solution penetrates, it readily forms adsorption bonds with aggregate particle surfaces, leading to aggregate detachment from the asphalt matrix and subsequent adhesion failure [83].
Rubber-modified asphalt; matrix asphalt	SiO_2_, CaO, Fe_2_O_3_, MgO	COMPASS	Isotropic displacement; diffusion coefficient; interfacial energy	The type of aggregate significantly influences the adhesion between the asphalt and aggregate. The greater the number of water molecules in contact, the more severe the damage to adhesive properties. Water molecules displace the asphalt originally present on the aggregate surface, leading to bond failure [84].
AAA-1 asphalt model	SiO_2_	COMPASS	Cohesive energy density; free volume fraction; interfacial adhesion energy	Owing to the differing effects of various metal ions on water molecules, sodium salts cause the most pronounced deterioration in the adhesion between asphalt and aggregate following erosion, whilst calcium and magnesium salts exert a lesser influence [35].
Matrix asphalt	SiO_2_, CaCO_3_	COMPASSII	Adhesion energy; debonding energy; degradation ratio (RAD); energy ratio (ER)	The adhesion between asphalt and aggregates diminishes with increasing chloride salt concentration, as chloride solutions spontaneously separate asphalt from aggregates. SiO_2_ exhibits superior adhesion to asphalt, thereby offering enhanced resistance to chloride salt erosion [85].
Matrix asphalt; bituminous slurry	SiO_2_, CaCO_3_	COMPASSIII	Radial distribution function; mean displacement; adhesion work and diffusion coefficient	Salt molecules compromise the adhesion properties at the degraded asphalt–aggregate interface, with their quantity exhibiting a positive correlation to the extent of interface degradation. Concurrently, decreasing temperatures weaken interfacial interactions, thereby adversely affecting adhesion performance [86].

**Table 3:** Laboratory testing of asphalt–aggregate bonding variations and key findings.

Material Type	Simulated Environment	Testing Method	Key Findings
Binder asphalt; basalt	Chlorides; sulphates; freeze–thaw cycles	Pull-out test; splitting test; microstructural analysis	Salt concentration and freeze–thaw cycles show a strong positive correlation with adhesion properties, with chloride salts having more destructive potential than sulfate salts [61].
70# base asphalt; basalt; granite	3.5% coarse salt solution simulating seawater; 0.5 MPa water pressure	Rheological properties test; moisture absorption rate test; pull-out test	Salt accelerates water ingress into structures, and the action of water pressure facilitates water reaching the adhesive interface, thereby accelerating the process of adhesive failure [58].
70# base asphalt; basalt; granite	3.5% coarse salt solution simulating seawater; hydrostatic pressure; wet–dry cycles; freeze–thaw cycles	Dynamic hydrostatic pressure/wet–dry cycle/freeze–thaw cycle erosion test; pull-out test	Water–salt erosion significantly degrades asphalt–aggregate adhesion, most markedly under dynamic water pressure. Moreover, salt–freeze–thaw or wet–dry cycles cause greater weakening than their water-only counterparts [62].
90# base asphalt, basalt; limestone powder	Chloride salts; sulphate salts; hydrostatic scouring	Dynamic water scouring/wet–dry cycling/freeze–thaw cycling; pull-out tests; normal-temperature/low-temperature splitting tests	Dynamic water scouring is more detrimental to adhesion than static erosion, with pull-off strength showing a significant negative correlation to freeze–thaw cycles. This weakening of the asphalt–aggregate bond heightens susceptibility to water damage [59].
High-viscosity modified asphalt; basalt; limestone mineral powder	Water; thermal-oxygen aging environment	Rotational viscosity testing; surface tension; FTIR testing	Although the combined effect of moisture and thermal oxidation initially increases asphalt viscosity, prolonged aging still degrades mechanical properties, primarily as a function of water content [60].
Base asphalt; SBS-modified asphalt; limestone	Salt spray environment (5% NaCl solution); freeze–thaw cycles	Freeze–thaw splitting test; freeze–thaw cycle splitting test	The tensile and splitting strength of asphalt mixtures declines under combined salt spray and freeze–thaw cycles, primarily driven by salt crystallization and ice expansion pressures within material voids, which severely degrade the asphalt–aggregate interface [63].
Cotton straw fiber; 90# base asphalt; basalt fiber	Composite salt solution (NaCl and Na_2_SO_4_); Na_2_SO_4_ solution; wet–dry and freeze–thaw cycles	Dry–wet–salt and freeze–thaw cycle splitting test; SEM testing; infrared spectroscopy testing	While salt accelerates the deterioration of the asphalt mixture, both cotton straw and chopped basalt fibers significantly enhance it. The degradation mechanism involves salt solution permeation during cyclic processes, which degrades the asphalt matrix and leads to bond failure [64].
SBS-modified asphalt; basalt; limestone powder; non-amine anti-stripping agent	NaCl solutions of varying concentrations; freeze–thaw cycles; wet–dry cycles; continuous immersion	Void content and splitting strength of mixture specimens	Although freeze–thaw/soaking cycles increase porosity and reduce splitting strength, most severely with a 10% NaCl solution after 10 cycles, basalt fibers effectively enhance the mechanical properties of the mixture [65].
SBS-modified asphalt; basalt; limestone	Slow-release anti-icing agent (primarily composed of NaCl)	Dynamic shear rheometric; low-temperature flexural rheology; contact angle measurement	Slow-release anti-icing agents enhance the hydrophobicity of asphalt, leading to a reduction in the adhesive work between asphalt and aggregates. Concurrently, the cohesive strength within the asphalt also diminishes to some extent. Ultimately, this results in the failure of the asphalt–aggregate bond, causing aggregate loss [26].
70# base asphalt; limestone; basalt; granite	3.5% industrial coarse salt solution; dynamic water pressure; static water erosion; wet–dry cycles	Water boiling method; pull-out test, CT scanning; SEM	Salt accelerates water ingress into the asphalt binder matrix. The osmotic pressure generated by the infiltrating salt solution, coupled with the expansive stress from crystallization, hastens the deterioration of adhesion and the decline in mechanical properties of the asphalt mixture [58,66].
70# base asphalt; SBS-modified asphalt; high-viscosity modified asphalt (TPS); limestone	Wet–dry and freeze–thaw cycles; salt solution	Dynamic shear rheometric; pull-out test; contact angle measurement	The surface energy of asphalt and the adhesion work between the asphalt and aggregate decrease with increasing salt–erosion cycles; high-surface-energy aggregate particles are more prone to detachment from the asphalt matrix via adsorbed water [67].
SBS composite modified asphalt (high-viscosity asphalt); basalt	Sodium chloride solution; sodium sulphate solution; composite salt solution; hydrostatic immersion; wet–dry cycle	Water-boiling method; tensile test; microstructural analysis	Both the surface tension and corrosive properties of seawater degrade the asphalt–aggregate interface. The corrosive effect of sulphate solutions is more pronounced than that of chloride and mixed salt solutions [68].
SBS-modified asphalt; limestone; manufactured sand	Sodium chloride solution; calcium chloride solution; freeze–thaw cycles; ultraviolet aging; salt–freeze cycles; ultraviolet aging	Micro-testing; freeze–thaw splitting test; rutting test	Salt corrosion hardens asphalt, whereby subsequent NaCl penetration and crystallization, combined with ice expansion stresses during freeze–thaw cycles and UV aging, induce interfacial failure and, consequently, pavement defects [69].

**Table 4:** Variation in tensile strength at the asphalt–aggregate interface under different test conditions.

Materials	Initial State (MPa)	Experimental Conditions	Final State (MPa)	Decrease Ratio (%)	Reference
90# base asphalt; basalt	Freeze–thaw cycle 7 times: 1.4	10% Na_2_SO_4_	1.05	25.00	[59]
		20% NaCl	0.95	32.14	
	Freeze–thaw cycle 28 times: 1.42	10% Na_2_SO_4_	0.98	30.99	
		20% NaCl	0.92	35.21	
SBS-modified asphalt; basalt	Soak in water for 8 h: 1.467	5% NaCl	1.301	11.11	[68]
		10% NaCl	1.480	−0.89	
		5% Na_2_SO_4_	1.091	25.63	
		5% composite (Cl^−^:SO_4_^2−^ = 7:1)	1.205	17.86	
	Soak in water for 6 days: 0.766	5% NaCl	0.656	14.36	
		10% NaCl	0.852	11.23	
		5% Na_2_SO_4_	0.541	29.37	
		5% composite (Cl^−^:SO_4_^2−^ = 7:1)	0.642	16.19	

**Table 5:** Variation in asphalt–aggregate interface ER_1_ index under different test conditions.

Materials	Initial State (ER_1_)	Experimental Conditions	Final State (ER_1_)	Decrease Ratio (%)	Reference
70# base asphalt; SBS-modified asphalt; limestone; compound salt solution (NaCl:Na_2_SO_4_ = 1:8)	Base asphalt: 0.96	Wet–dry freeze–thaw cycle 5 times	0.85	11.46	[67]
		Wet–dry freeze–thaw cycle 10 times	0.74	22.92	
		Wet–dry freeze–thaw cycle 15 times	0.61	36.46	
		Wet–dry freeze–thaw cycle 20 times	0.56	41.67	
	SBS-modified asphalt: 1.21	Wet–dry freeze–thaw cycle 5 times	1.06	12.40	
		Wet–dry freeze–thaw cycle 10 times	0.89	26.45	
		Wet–dry freeze–thaw cycle 15 times	0.77	36.36	
		Wet–dry freeze–thaw cycle 20 times	0.69	42.98	

**Table 6:** Variation in asphalt–aggregate interface ER_2_ index under different test conditions.

Materials	Initial State (ER_2_)	Experimental Conditions	Final State (ER_2_)		Decrease Ratio (%)		Reference
			L	B			
Base asphalt; SBS-modified asphalt; limestone; basalt	Base asphalt-L: 2.359 Base asphalt-B: 1.7852	Wet–dry freeze–thaw cycle 8 times (NaCl)	1.8715	1.3769	20.67	22.87	[70]
		Wet–dry freeze–thaw cycle 15 times (NaCl)	1.5546	1.0852	34.10	39.21	
		Wet–dry freeze–thaw cycle 25 times (NaCl)	1.3041	0.9361	44.72	47.56	
		Wet–dry cycle 25 times and freeze–thaw cycle 8 times (NaCl)	1.1094	0.7703	52.97	56.85	
		Wet–dry freeze–thaw cycle 8 times (Na_2_SO_4_)	1.5791	1.1459	33.06	35.81	
		Wet–dry freeze–thaw cycle 15 times (Na_2_SO_4_)	1.2846	0.9104	45.54	49.00	
		Wet–dry freeze–thaw cycle 25 times (Na_2_SO_4_)	0.9013	0.7144	61.79	59.98	
		Wet–dry cycle 25 times and freeze–thaw cycle 8 times (Na_2_SO_4_)	0.6867	0.4575	70.89	74.37	
	SBS-modified asphalt-L: 3.0904 SBS-modified asphalt-B: 2.1104	Wet–dry freeze–thaw cycle 8 times (NaCl)	2.5324	1.7998	18.06	14.72	
		Wet–dry freeze–thaw cycle 15 times (NaCl)	2.3490	1.6885	23.99	19.99	
		Wet–dry freeze–thaw cycle 25 times (NaCl)	2.0184	1.4799	34.69	29.88	
		Wet–dry cycle 25 times and freeze–thaw cycle 8 times (NaCl)	1.6392	1.1494	46.96	45.54	
		Wet–dry freeze–thaw cycle 8 times (Na_2_SO_4_)	2.0704	1.4857	33.01	29.60	
		Wet–dry freeze–thaw cycle 15 times (Na_2_SO_4_)	1.8851	1.3192	39.00	37.49	
		Wet–dry freeze–thaw cycle 25 times (Na_2_SO_4_)	1.6694	1.2367	45.98	41.40	
		Wet–dry cycle 25 times and freeze–thaw cycle 8 times (Na_2_SO_4_)	1.4220	1.0973	55.7	49.0	

## Data Availability

No new data were created or analyzed in the study. Data sharing is not applicable to this article.
